# Correction: *Drosophila* PDGF/VEGF signaling from muscles to hepatocyte-like cells protects against obesity

**DOI:** 10.7554/eLife.66685

**Published:** 2021-01-21

**Authors:** Arpan C Ghosh, Sudhir Gopal Tattikota, Yifang Liu, Aram Comjean, Yanhui Hu, Victor Barrera, Shannan J Ho Sui, Norbert Perrimon

Ghosh AC, Tattikota SG, Liu Y, Comjean A, Hu Y, Barrera V, Sui SJH, Perrimon N. 2020. *Drosophila* PDGF/VEGF signaling from muscles to hepatocyte-like cells protects against obesity. *eLife* **9**:e56969. doi: 10.7554/eLife.56969.

Published 27, October 2020

While preparing Figure 5 for the manuscript, three Thin-layer Chromatography lanes in Figure 5E got inadvertently duplicated, a mistake that was brought to our notice by a reader of the published article. While revisiting our original TLC blots, we realized that in Figure 5C the labelling of our experimental TLC lanes were unintentionally swapped. Additionally, TLC data shown in Figure 5B, C, D and E were assembled by splicing out irrelevant lanes from the plates from which each of those figures were prepared, however, the junctions were not demarcated. The corrections do not affect or change the corresponding quantifications for the TLC lanes in Figure 5. Neither do they change or affect any of the conclusions of the manuscript.

The corrected text in the figure legend of Figure 5 (change is underlined):

Figure 5: Muscle to oenocyte Pvf1 signaling suppresses lipid synthesis.

(A) Starvation resistance of adult male flies on 1% sucrose and 0.8% agar food. Males with muscle-specific knockdown of *pvf1* (*mus^ts^>pvf1-i1*) and oenocyte-specific loss of PvR signaling (*oeno^ts^*>*pvr^DN^*) were significantly more resistant to starvation compared to respective controls flies (*mus^ts^= muscle-Gal4^Gal80ts^* and *UAS-pvr^DN^/+*). N = 100, Log rank (Mantel-Cox) test, *p≤0.0001* for both comparisons, error bars = SEM.

(B) A representative TLC plate and corresponding quantification of the TLC bands showing rate of lipid mobilization in control (*mus^ts^*) and flies with muscle-specific loss of *pvf1* (*mus^ts^>pvf1-i1*). N = 4, Multiple Student t-test, error bars = SEM. Note: The control and experimental TLC bands were rearranged for representation from the same TLC plate. Junctions are marked with a dotted line.

(C) A representative TLC plate and corresponding quantification of the TLC bands showing rate of lipid mobilization in control (*oeno^ts^=oenocyte-Gal4^Gal80ts^*) and flies with oenocyte-specific loss of TOR signaling (*oeno^ts^>tsc1,tsc2*) and PvR signaling (*oeno^ts^>pvr^DN^*). N = 4, Multiple Student t-test, error bars = SEM. Note: The control and experimental TLC bands were rearranged for representation from the same TLC plate. Junctions are marked with a dotted line.

(D) A representative TLC plate and corresponding quantification of the TLC bands showing rate of lipid synthesis and incorporation from [U-^14^C]-Sucrose in control (*mus*^ts^) and flies with muscle-specific loss of *pvf1* (*mus^ts^>pvf1-i1*). Flies lacking Pvf1 in the muscle show a significantly faster rate of lipid incorporation compared to control animals. N = 4, ** denotes *p≤0.01* at 72 hr on hot food. Multiple student t-test, error bars = SEM. Note: The control and experimental TLC bands were rearranged for representation from the same TLC plate. Junctions are marked with a dotted line.

(E) A representative TLC plate and corresponding quantification of the TLC bands showing rate of lipid synthesis and incorporation from [U-^14^C]-Sucrose in control flies (*oeno^ts^*) and flies with oenocyte-specific loss of TOR signaling (*oeno^ts^>tsc1,tsc2*) and PvR signaling (*oeno^ts^>pvr^DN^*). Flies lacking TOR or PvR signaling in the oenocytes show a significantly faster rate of lipid incorporation compared to control animals. N = 4, *** denotes *p≤0.001* at 72 hr on hot food. Multiple student t-test, error bars = SEM. Note: The control and experimental TLC bands were rearranged for representation from the same TLC plate. Junctions are marked with a dotted line.

(F) Expression level of key lipid synthesis genes in control (*oeno^ts^*) and flies with oenocyte-specific loss of TOR signaling (*oeno^ts^>tsc1,tsc2*). Only oenocyte-specific fatty acid synthases, *fasn2* and *fasn3*, show a significant reduction in expression in the experimental flies. N = 4, One-Way Anova followed by Tukey’s HSD test, **** denotes *p*≤*0.0001*, error bars = SEM.

The corrected Figure 5 is shown here:

**Figure 1:**
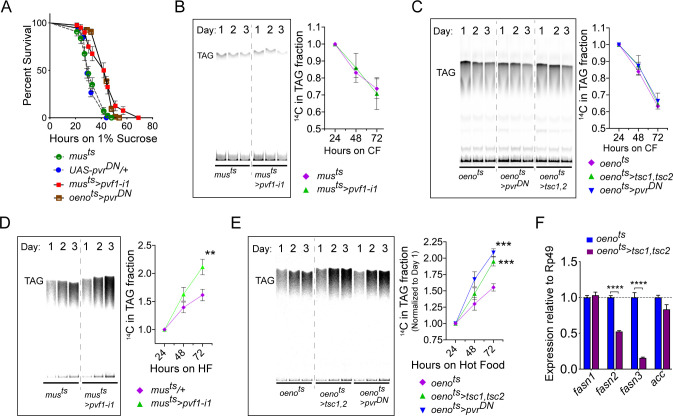


The originally published Figure 5 is also shown here for reference:

**Figure 2:**
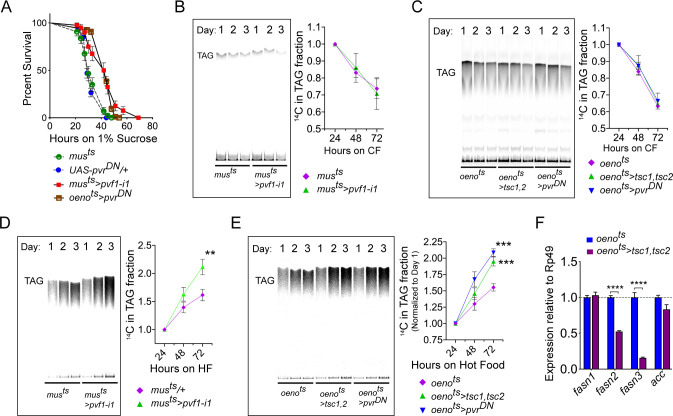


The article has been corrected accordingly. We apologize for the error.

