# Supplementary material for: Drosophila PDGF/VEGF signaling from muscles to hepatocyte-like cells protects against obesity
Source: eLife. 2020 Oct 27;9:e56969. doi: 10.7554/eLife.56969 (PMC7752135; doi:10.7554/eLife.56969)
Supplement: Transparent reporting form [file elife-56969-transrepform.docx]

***eLife’s* transparent reporting form**

We encourage authors to provide detailed information *within their submission* to facilitate the interpretation and replication of experiments. Authors can upload supporting documentation to indicate the use of appropriate reporting guidelines for health-related research (see [EQUATOR Network](http://www.equator-network.org/%20)), life science research (see the [BioSharing Information Resource](https://biosharing.org/)), or the [ARRIVE guidelines](http://www.plosbiology.org/article/info:doi/10.1371/journal.pbio.1000412) for reporting work involving animal research. Where applicable, authors should refer to any relevant reporting standards documents in this form.

If you have any questions, please consult our Journal Policies and/or contact us: [editorial@elifesciences.org](mailto:editorial@elifesciences.org).

**Sample-size estimation**

- You should state whether an appropriate sample size was computed when the study was being designed
- You should state the statistical method of sample size computation and any required assumptions
- If no explicit power analysis was used, you should describe how you decided what sample (replicate) size (number) to use

Please outline where this information can be found within the submission (e.g., sections or figure legends), or explain why this information doesn’t apply to your submission:

We did not use any sample size computation nor power analysis for whole animal TAG assays, quantification of fluorescent images and radio-isotope incorporation assays. We decided to have a sample size of 6 replicates for all TAG assays based on previous publications that show that a sample size of 4-6 replicates is sufficient for detecting changes in whole animal TAG levels caused by genetic manipulations or changes in food content. Sample sizes for imaging-based quantification of lipid droplets and p4EBP staining was decided based on limitations placed by the practicality of how many samples could be dissected for each of the experiments. We used single images from 5-6 individuals for all lipid droplet size measurements. For p4EBP staining use used 6-10 individuals with only a single oenocyte cluster imaged per individual. We used 4 biological replicates for all TLC assays and qRT-PCR assays. Once again the limit was decided based on the number of animals we could rear on radiolabeled food for a given experiment. For single nuclei sequencing experiments, we decided to have 2 biological replicates and adequate number of single nuclei (~15K) in total.

**Replicates**

- You should report how often each experiment was performed
- You should include a definition of biological versus technical replication
- The data obtained should be provided and sufficient information should be provided to indicate the number of independent biological and/or technical replicates
- If you encountered any outliers, you should describe how these were handled
- Criteria for exclusion/inclusion of data should be clearly stated
- High-throughput sequence data should be uploaded before submission, with a private link for reviewers provided (these are available from both GEO and ArrayExpress)

Please outline where this information can be found within the submission (e.g., sections or figure legends), or explain why this information doesn’t apply to your submission:

**TAG assays (Fig. 1, Fig. 2):** A single data point consisted of TAG measurements from homogenates of 8 males. 6 such samples (considered biological replicates) were used per analysis. All TAG assay experiments were repeated 2-3 times and only outcomes that remained consistent are reported. We did not pool measurements from different experimental replicates since there was no way to normalize for batch effects.

**Information on number of replicates, statistical method used, and p-values is presented in the figure legends.**

**Measurements of Lipid droplet size (Fig. 1, Fig. 3, Fig. 4):** Individual animals were considered biological replicates. For each data point a total of about 10 images acquired from one of the adipose tissue lobes of each animal. We counted hundreds of lipid droplets per image and the mean lipid droplet size from these images is reported as a single data point per animal. 5-6 biological replicates were used to determine mean lipid droplet size.  **Information on number of replicates, statistical method used, and p-values is presented in the figure legends.**

**Measurement of p4EBP immunostaining intensities in oenocytes (Fig. 4):** Individual animals were considered biological replicates and a single image was obtained per biological replicate. 6-10 biological replicates were used per experiment. **Information on number of replicates, statistical method used, and p-values is presented in the figure legends.**

**Starvation resistance assay (Fig. 5):** We started with 100 individual per genotype to generate the survivability curve. Experiments were performed twice but data from only one such experiment is provided. We only reported observations that were consistent between the two experiments.

**Information on number of replicates, statistical method used, and p-values is presented in the figure legends.**

**TLC based 14C incorporation assays (Fig 5):** 23 adult males were used per sample point. 4 such sample points (biological replicates) were generated by 4 independent experiments. **Information on number of replicates, statistical method used, and p-values is presented in the figure legends.**

**qRT-PCR assay (Fig. 5, Fig. 6):** Total RNA was extracted from either 10 abdominal cuticles (FB samples, Figure 5F) or 10 thoraxes (Muscle samples, Figure 6A) was used to generate a single sample point and is considered a biological replicate. 4 Biological replicates were used. **Information on number of replicates, statistical method used, and p-values is presented in the figure legends.**

**Statistical reporting**

- Statistical analysis methods should be described and justified
- Raw data should be presented in figures whenever informative to do so (typically when N per group is less than 10)
- For each experiment, you should identify the statistical tests used, exact values of N, definitions of center, methods of multiple test correction, and dispersion and precision measures (e.g., mean, median, SD, SEM, confidence intervals; and, for the major substantive results, a measure of effect size (e.g., Pearson's r, Cohen's d)
- Report exact p-values wherever possible alongside the summary statistics and 95% confidence intervals. These should be reported for all key questions and not only when the p-value is less than 0.05.

Please outline where this information can be found within the submission (e.g., sections or figure legends), or explain why this information doesn’t apply to your submission:

All the statistics for the replicates mentioned above were performed using Prism8. The “Statistics” section in Methods describes the statistical methods used and the detailed information with regards to statistical methods used to determine significant and the error bars (SEM) can be found in the figure legends wherever necessary. p values are also mentioned alongside in the figure legends, wherever necessary.

(For large datasets, or papers with a very large number of statistical tests, you may upload a single table file with tests, Ns, etc., with reference to sections in the manuscript.)

**Group allocation**

- Indicate how samples were allocated into experimental groups (in the case of clinical studies, please specify allocation to treatment method); if randomization was used, please also state if restricted randomization was applied
- Indicate if masking was used during group allocation, data collection and/or data analysis

Please outline where this information can be found within the submission (e.g., sections or figure legends), or explain why this information doesn’t apply to your submission:

N.A.

**Additional data files (“source data”)**

- We encourage you to upload relevant additional data files, such as numerical data that are represented as a graph in a figure, or as a summary table
- Where provided, these should be in the most useful format, and they can be uploaded as “Source data” files linked to a main figure or table
- Include model definition files including the full list of parameters used
- Include code used for data analysis (e.g., R, MatLab)
- Avoid stating that data files are “available upon request”

Please indicate the figures or tables for which source data files have been provided:

Source data is provided for Figure 2 B and Figure 2C in Table S2 and Table S3 respectively.
